# Climate-driven spatial distribution and ecogeographical analysis of Cyperaceae using GIS and remote sensing

**DOI:** 10.3897/BDJ.14.e194618

**Published:** 2026-10-28

**Authors:** Sayyara Jamshid Ibadullayeva, Narmin Ilham Sadigova, Zulfiyya Jalal Mammadova, Nuri Vagif Movsumova, Gulnara Shirali Shiraliyeva, Samira Farman Khudaverdiyeva

**Affiliations:** 1 Institute of Botany, Ministry of Science and Education of the Republic of Azerbaijan, Baku, Azerbaijan Institute of Botany, Ministry of Science and Education of the Republic of Azerbaijan Baku Azerbaijan; 2 Baku State University, Faculty of Biology, Department of "Botany and Plant Physiology", Baku, Azerbaijan Baku State University, Faculty of Biology, Department of "Botany and Plant Physiology" Baku Azerbaijan

**Keywords:** Azerbaijan, climate change, Cyperaceae, ecogeography, GIS, RS, vegetation distribution

## Abstract

The research is dedicated to the study of the ecogeographic distribution of the Cyperaceae Juss. family in Azerbaijan, related to the dynamic changes occurring in plant communities formed under the influence of landscape diversity and climatic factors through RS (Remote Sensing) and GIS. Based on the results of the study, 115 taxa consisting of 14 genera belonging to the Cyperaceae family were identified. These taxa are widespread in eight of the eleven main landscape types of Azerbaijan. According to their ecological requirements for humidity, 58% of species belong to the mesophytic, 21% to the hygromesophytic, 16% to the hygrophytic and 5% to the xerophytic ecological groups. Representatives of the family are mainly observed in marshy, meadow, and coastal zones, which indicates their high ecological sensitivity to the hydrological regime. WorldClim (average annual temperature and precipitation indicators for 2020 and 2024) and OpenTopography (Digital Elevation Model) data were used for the research, and the analyses were conducted using ArcGIS Pro software. The aim was to assess the spatial distribution of temperature and precipitation and to examine their interannual variation in relation to Cyperaceae plant communities during 2020–2024. A comparison of data from the WorldClim database shows that, between 2020 and 2024, the minimum temperature increased from −10.2°C to −8.8°C, the maximum temperature increased from 7.75°C to 8.13°C and annual precipitation decreased, albeit partially. These interannual climatic differences may potentially influence moisture-dependent habitats and Cyperaceae plant communities, particularly in regions where water availability is already limited. The integration of remote sensing, spatial data analysis and climate data provides a useful framework for assessing potential relationships between climatic variation, vegetation distribution and biodiversity.

## Introduction

Cyperaceae or sedge family, one of the large monocotyledonous families, ranking third after the Poaceae and Orchidaceae Juss., also includes a large number of genera and species (Jaafa 2025). Some of them have economic importance as they are a source of food and fuel and some of them are used in various industries, such as textiles, perfumes and building materials (Simpson and Inglis 2001). Traditionally, there are species in the family that are used as a treatment for various diseases such as: skin infection, urinary disorders and digestive disorders (Chauhan et al. 2024), some of them being an indicator of the deterioration of wetland environments (Muasya et al. 2009). Members of this family are widely distributed throughout the world, particularly in humid temperate regions and sub-polar environments. Cyperaceae family is one of the most important ecological components of the meadow and wetland vegetation of Azerbaijan. Species belonging to this family act as bioindicators of hydrological balance and soil moisture stability. Given the strong dependence of many Cyperaceae species on soil moisture and hydrological conditions, variations in temperature and precipitation may influence the distribution and ecological stability of their habitats. Representatives of genera, such as *Carex* L., *Cyperus* L., *Schoenoplectus* (Rchb.), *Eleocharis* R.Br., *Scirpus* Tourn. ex L. included in the family Cyperaceae, are widespread species usually found in humid, swampy and semi-swampy areas, in river valleys, along the edges of standing waters and channels and on humid mountain slopes. These plants are very sensitive to soil moisture and the hydrological regime. Thus, the fact that 58% of the 115 taxa studied are mesophytes, 21% are hygromesophytes and 16% are hygrophytes indicates that these plants can be considered phytoindicators of humidity.

Effects of climate change are already being observed on a wide range of ecosystems and species in all regions of the world (Rosenzweig et al. 2007). Specifically, temperature and precipitation are both expected to shift globally and their separate and interactive effects will likely affect ecosystems differentially depending on current temperature, precipitation regimes and other biotic and environmental factors (Cowles et al. 2018). Although extensive taxonomic studies have been conducted on the flora of Azerbaijan, the ecogeographic distribution and variability of the Cyperaceae family in various landscapes of Azerbaijan have not been fully studied using modern GIS (Geographic Information Systems) and remote sensing methods (Ibadullayeva et al. 2026). The main objectives of this study are to analyse the ecogeographic distribution of Cyperaceae species, soil and landscape relationships, classify species into ecological groups according to water and light regimes and assess the potential relevance of temperature and precipitation variation to Cyperaceae plant communities during 2020–2024 using ArcGIS Pro software, based on WorldClim and OpenTopography data.

## Material and methods

**Research area**

**Figure 1.**
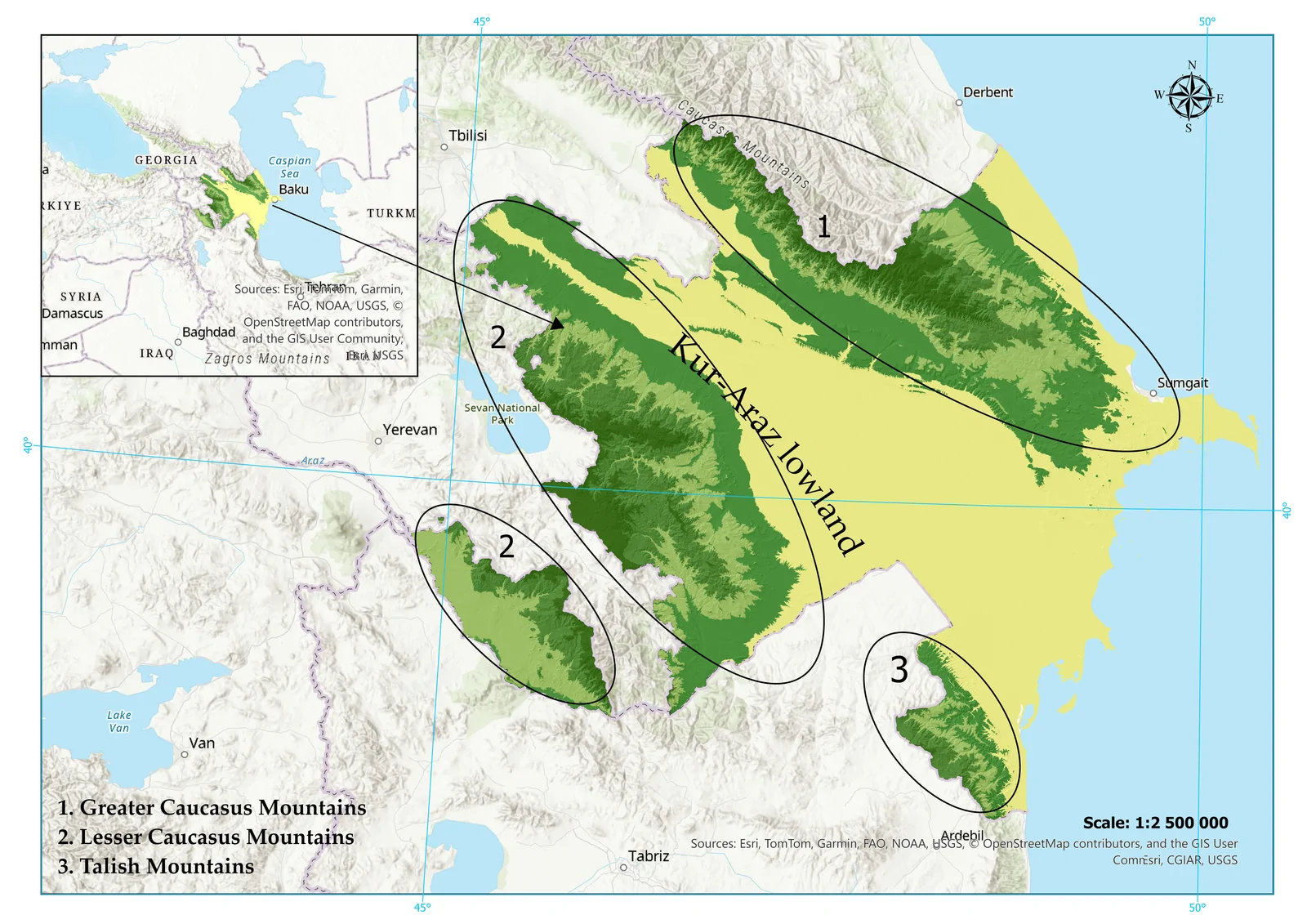
Azerbaijan's location on the world map (please include the world map showing where Azerbaijan is located).

The main geomorphological structures of the Republic of Azerbaijan are the Greater Caucasus, the Lesser Caucasus (with the Karabakh Plateau) and the Talysh Mountains. They cover the Kura-Araz lowland from the north, west and southeast. Despite the fact that the plains cover a large area in the Republic, they are surrounded by mountain ranges. The Greater Caucasus, Lesser Caucasus and Talysh Mountains in the country occupy 2/3 of the territory (Fig. 1). Azerbaijan is located between 38° and 41° north latitude and 44° and 50° east longitude (Bayramov 2023). This geomorphological diversity creates rich ecological conditions for representatives of the Cyperaceae family. The highest point in Azerbaijan is Mount Baz arduzu (4,485 m) in the Greater Caucasus and the lowest point is the level of the Caspian Sea (-28 m). The country is located at the crossroads of Europe and Asia and has a unique geopolitical and geographical position [https://namazerbaijan.org/about-azerbaijan-geography].

**Data sources**

General information on Cyperaceae taxa distributed in Azerbaijan was compiled from classical and modern scientific sources, including current taxonomic information from World Flora Online (2026). During the research, the flora conspectus of the species and the main ecosystems were based on the works “Flora of Azerbaijan” (1952), the landscape types of plants on the works “National Encyclopedia of Azerbaijan, Landscapes” (Budagov 2007), the soil types on the works “Physical Geography of Azerbaijan” (Museibov 1998) and “Fundamentals of Soil Science and Soil Geography” (Mammadov 2007), the water regime on the works “General Ecology” (Chernova and Bylova 2004) and the light regime on the works “Ecology and Environmental Protection” (Mammadov and Mahmudov 2005).

**Geographic Information Systems-based methods**

One of the areas of application of Geographic Information Systems is the analysis of data obtained during environmental monitoring. The effectiveness of environmental monitoring relies on the accurate collection, summary and analysis of diverse data (Parra 2022). Currently, there are many software products that perform the functions of Geographic Information Systems, the most modern and widespread of which is the ArcGIS Pro programme. ArcGIS Pro is a licensed functional, desktop geoinformation system. The programme has extensive capabilities and supports numerous vectors, raster formats and databases (Huang et al. 2021). WorldClim is a reliable data source that can provide ready information for current and future scenarios. In the present study, the climatic analysis was restricted to the 2020–2024 study period. The analysis was designed to examine the spatial distribution and interannual variation of temperature and precipitation within this selected period rather than to establish a long-term climatological normal or quantify long-term climate change. Accordingly, differences observed between the analysed years are interpreted as interannual climatic variation. The main application area is to investigate the ranges of native and invasive, as well as harmful species and to model the results, based on climate-related variations (https://www.worldclim.org/data/worldclim21.html, Fick and Hijmans (2017), Poggio et al. (2018)Portal (2023) ).The OpenTopography scientific portal (OpenTopography (2026); https://portal.opentopography.org/dataCatalog) is an online scientific portal that provides access to a high-quality topographic database. All three methods are methods of information systems, data management, software and its engineering (Nandigam et al. 2017). During the preparation of this manuscript, the authors used Claude (Anthropic) to assist with English language editing and academic writing improvement. All scientific content, data, analyses and conclusions are solely the responsibility of the authors.

## Results

**Floristic analysis**

At the beginning of the study, general information on Cyperaceae taxa, distributed in Azerbaijan, was collected. When comparing this data with the latest results of modern references, such as WFO [https://www.worldfloraonline.org/search?query=cyperaceae], it can be seen that species specific to genera, such as *Juncellus* C.B.Clarke (= *Cyperus* L.), *Acorellus* Palla ex Kneuck.(= *Cyperus* L.), *Pycreus* P.Beauv.(= *Cyperus* L.), *Torulinium* Desv.(= *Cyperus* L.), have been transferred to the genus *Cyperus* L. according to modern classification and species belonging to genera, such as *Elyna* Schrad.(= Carex L.), *Cobresia* Will (= *Carex* L.), to the genus *Carex* L. At the same time, when applying to WFO (World Flora Online 2026), it was determined that 20 of the species currently included in the family have been transferred to subspecies with synonymous names and two to variations.

**Ecogeographic analysis**

**Figure 2.**
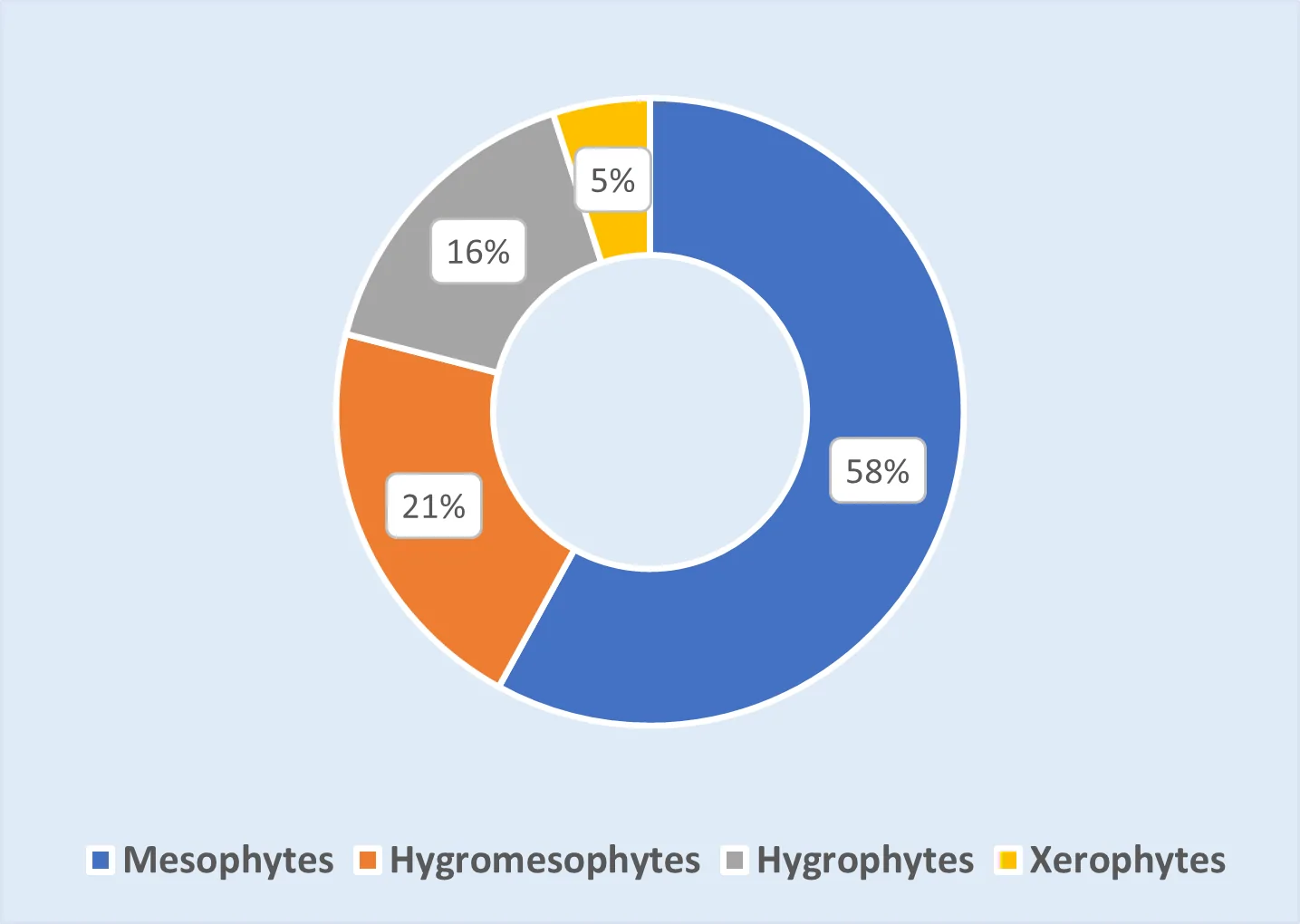
Percentage of ecological groups according to their water regime.

When analysing the water regime of representatives of the Cyperaceae family, it was found that, of the 115 taxa found in Azerbaijan, 67 (58%) are mesophytes, 24 (21%) hygromesophytes, 18 (16%) hygrophytes and six (5%) xerophytes (Fig. 2). Thus, mesophytic species are species that are mainly distributed in meadows, forests and shrubs, in humid-sandy areas, in valleys and around rivers and water channels. These are considered the largest group in terms of percentage. Examples of mesophytic species include *Cyperus rotundus* L., *Mariscus cyri* Grossh, *Blysmus compressus* (L.) Panz.ex Link, *Scirpus silvaticus* L., *Fimbristylis dichotoma* (L.) Vahl and *Carex remota* L.. Hygromesophytic species are found not only in swamps, but also around rivers and channels, as well as rice plantations and in moist meadows. Examples of such species are *Cyperus badius* Poir. (= *C. longus subsp. badius* Desf. B&L), *Cyperus aureus* Ten. (= *C. esculentus* L.), *Eriophorum latifolium* Hoppe and *Carex compacta* Lam (= *Carex vulpina* L.). The third group we analysed according to water regime is hygrophytes. Hygrophytes are species that are widespread mainly in swamps and rice plantations, examples of which include *Cyperus difformis* L., *Cyperus glaber* L., *Eleocharis euuniglumis* Zinserl, *Carex vesicaria* L., *Carex caespitosa* L. etc. Amongst the species of the Cyperaceae Juss. family widespread in Azerbaijan, the group with the lowest percentage of water regime is xerophytes. Xerophytes are species found mainly on stony-gravelly screes, dry slopes and sandy-gravelly dry areas. Examples of these include *Carex supina* Wahlenb, *Carex colchica* J.Gay, *Carex pachystylis* J.Gay etc.

**Figure 3.**
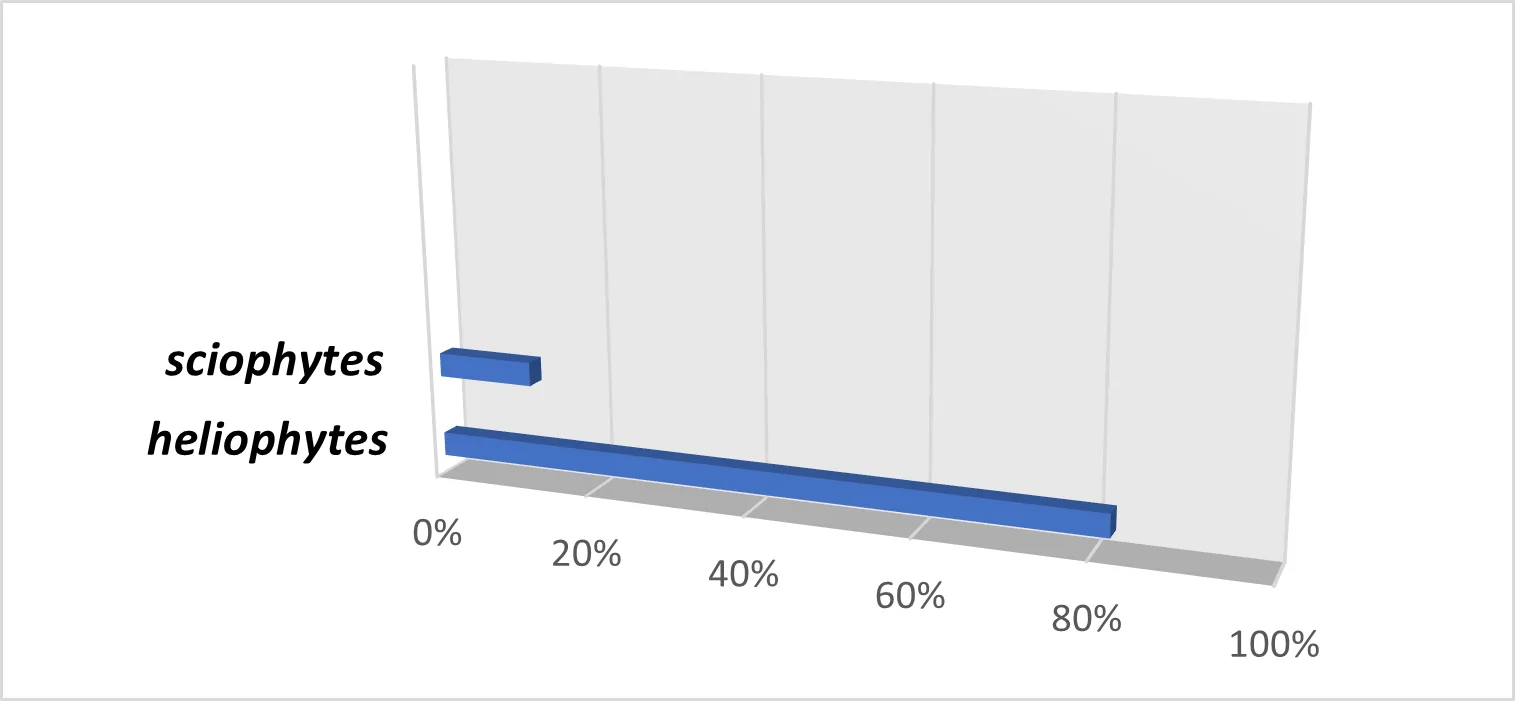
Percentage of ecological groups according to their light regime.

According to the light regime, plants are divided into two ecological groups: heliophytes and sciophytes. Light-demanding plants, or heliophytes, include plants of open areas and constantly lit places (meadows, slopes, semi-desert areas). These plants are rarely found in the forest zone. Heliophytes predominate amongst the representatives of the Cyperaceae family distributed in Azerbaijan. Thus, 94 out of 115 taxa (82%) are light-demanding plants. Shade-loving plants, or sciophytes, are plants of the lower tier of shady forests. These plants do not tolerate strong direct sunlight well. The studied 21 taxa (18%) of the family are sciophytic plants, distributed mainly in shady forests and thickets (Fig. 3). Examples of these include *Eleocharis intersita* Zinserl. (= *E. palustris subsp. palustris*), *Carex remota* L., *C. divulsa* Stokes, *C. digitata* L., *C. phyllostachys* C.A.M., *Scirpus silvaticus* L. etc.

**Landscape - soil relations**

Scientific research has shown that the landscapes and soil types in which species are distributed interact with one another and these factors collectively form a specific ecosystem. Thus:

- turfy mountain-meadow soils are characteristic of the alpine and subalpine meadow-steppe landscapes of the high mountains and the species found in this specific environment are usually species adapted to the alpine and subalpine meadow ecosystem.
- the soils typical of the broadleaf forest and post-forest meadow-shrub landscape of the middle highlands are brown mountain-forest, sedimentary-carbonate mountain-forest, typical and washed mountain-forest soils, where the species distributed are mainly adapted to humid-shady forests, shrubs, forest meadows and swampy meadows and the ecosystem typical of the surroundings of rivers and lakes.
- the soils characteristic of the mountain-xerophyte landscape of the middle highlands are mountain-chestnut and brown mountain-forest soils. The ecosystem created by these two factors is mainly dry-grassy valleys and rocky-gravelly scree.
- In the steppe and partly forest-steppe landscape of the low mountains, mountain dark chestnut, mountain grey-brown and brown mountain-forest soils are widespread. These elements form ecosystems consisting of swamps, humid areas and shady forests.
- It creates dry, sandy ecosystems on brown, light brown and grey-brown soils typical of the semi-desert landscape of the lower mountain belt.
- In the broad-leaved forest landscape of the foothills, carbonate and typical brown mountain-forest and podzolised mountain-yellow soils are widespread. It consists of humid areas, forests, shrubs and wetlands, which create appropriate landscape-soil-climatic factors.
- alluvial meadow-forest, alluvial meadow and meadow-forest soils and yellow podzolic and gley podzolic soils are common in the forest-meadow landscapes of the lowland-plains. Ecosystems are formed here mainly created by swampy meadows, moist-sandy, clayey-saltworts and moist river banks.
- Grey-meadow, grey and grey-brown soils are widespread in the meadow-swamp subtype of semi-desert landscapes of lowlands and intermountain plains, where ecosystems formed by silty-marshy, marshy meadows and moist, silty and sandy riverbanks are characteristic.

**Figure 4.**
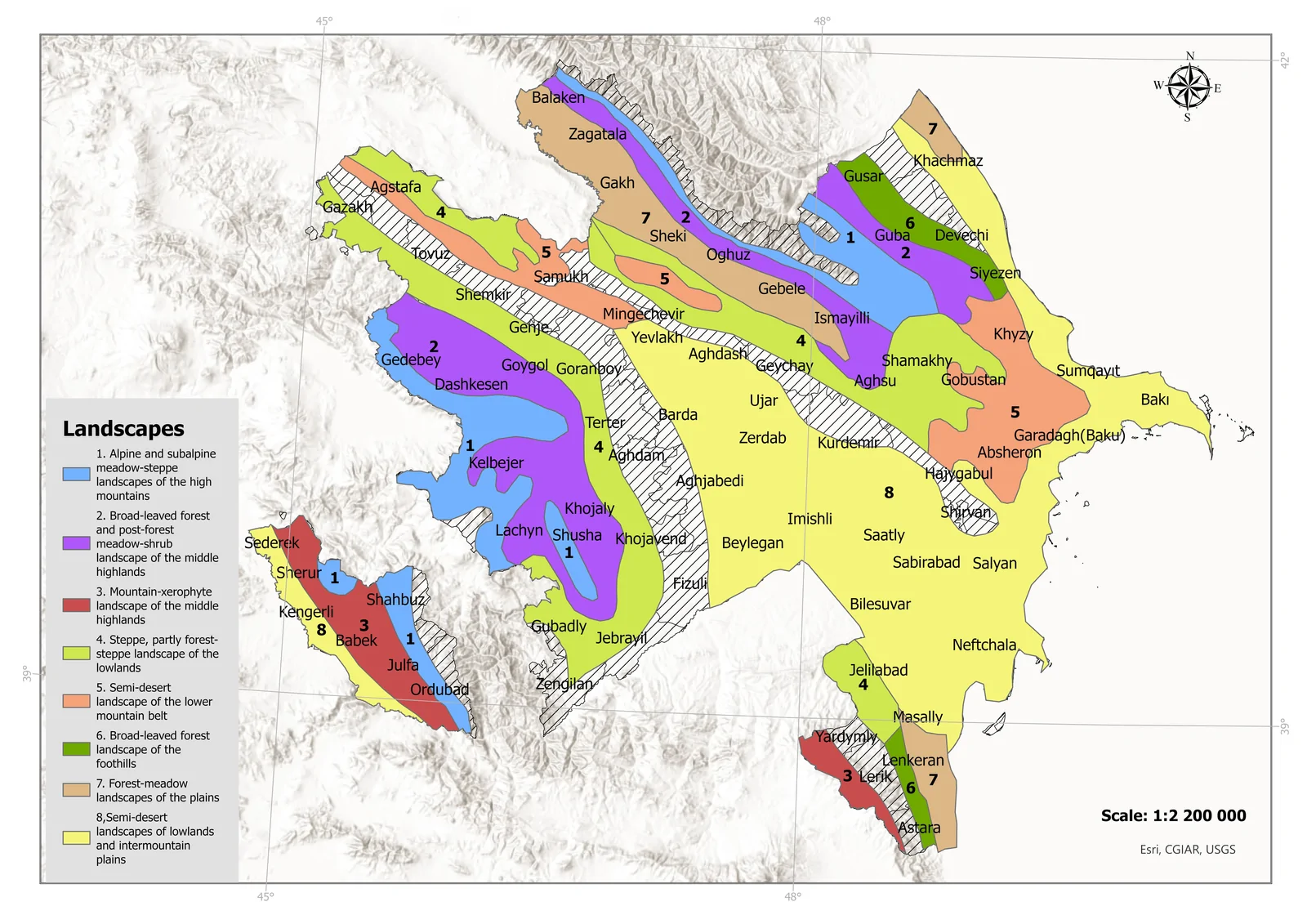
Main landscapes where the Cyperaceae Juss. family is distributed.

Based on the above, a GIS map of the landscape types where representatives of the Cyperaceae Juss. family are distributed in Azerbaijan was developed (Fig. 4).

As can be seen from the map, representatives of the Cyperaceae family are widely distributed in eight of the eleven landscape types existing in Azerbaijan (Budagov 2007).

The landscapes where species belonging to the studied family are not found at all include mainly the nival and partially nival-glacial landscapes of the high mountains, the semi-desert landscapes of the low mountains and the dry-steppe landscapes of the plains.

**The impact of climate change on the dynamics of Cyperaceae plant communities**

Representatives of this family are considered an ecological group that is highly sensitive to temperature changes Sousa et al. (2025). For this reason, in the context of increasing temperatures and decreasing precipitation, noticeable changes may occur in the dynamics of plant communities formed by representatives of this family. Two main environmental factors, such as rising temperatures and decreasing precipitation, lead to increased evaporation, reduced soil moisture and, simultaneously, negative changes in the water balance, soil drying out and a reduction in the area of wetlands. These processes negatively affect the living conditions of hydrophilic species, such as Cyperaceae, lead to the replacement of phytocenoses with drought-adapted species and make the possibility of vegetation zones shifting very high. These changes may negatively impact ecosystem stability and biodiversity in the future.

**Figure 5.**
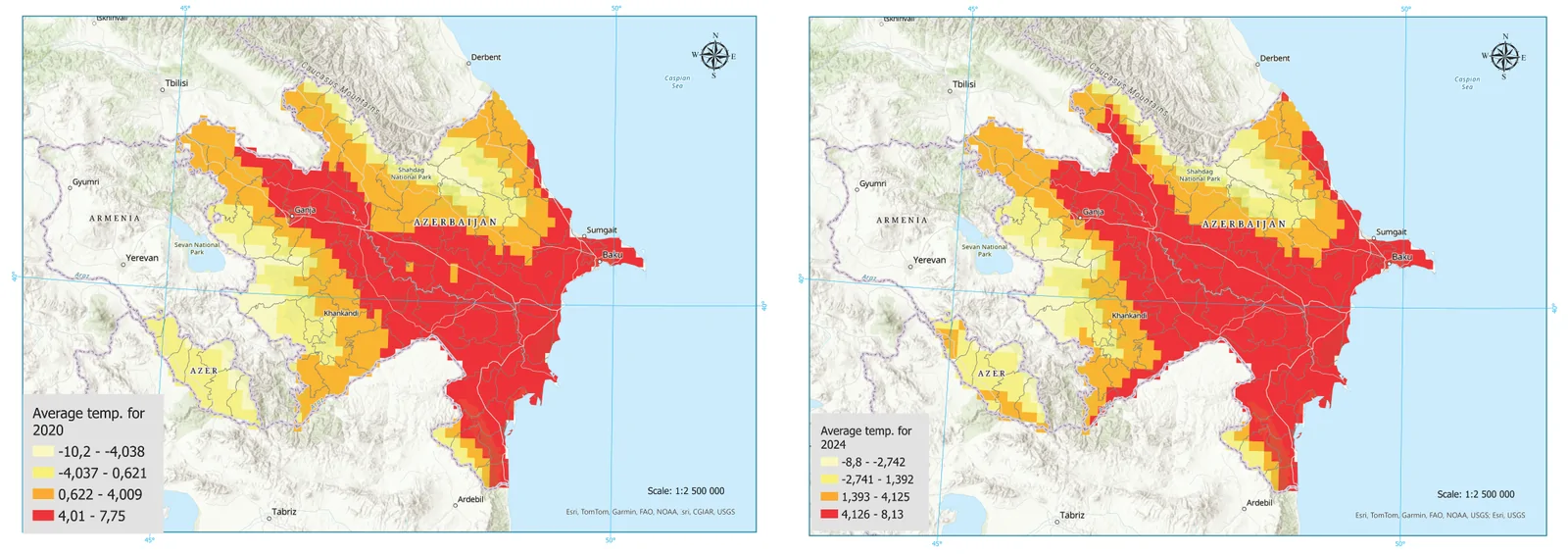
Average annual temperature indicators for the years 2020-2024.

**Figure 6.**
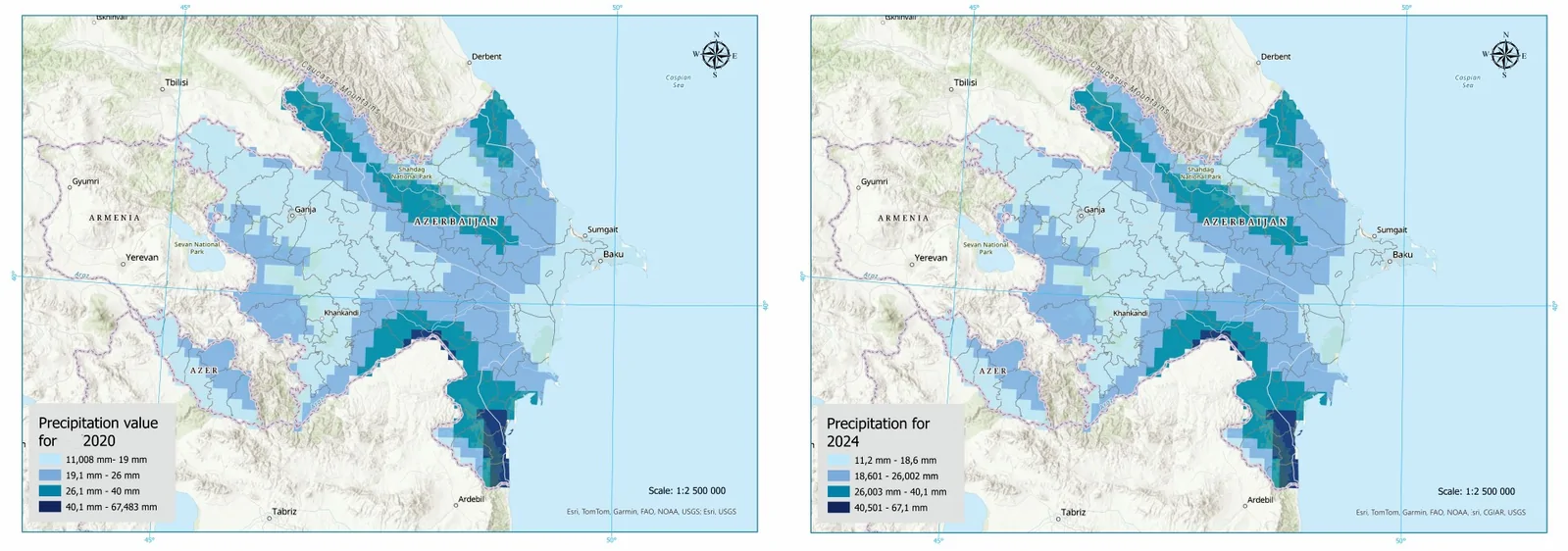
Spatial distribution of precipitation for the years 2020 and 2024.

The spatial distribution of temperature and precipitation in the maps developed by us was analysed, based on the DEM (Digital Elevation Model) obtained from the WorldClim database and processed in ArcGIS Pro. The maps compare climate indicators for the years 2020 and 2024. The developed maps illustrate the spatial differences in temperature and precipitation between the analysed years (Figs 5, 6).

Fig. 5 reveals that annual average temperature analysis was carried out separately for Azerbaijan for the years 2020 and 2024. The areas depicted here in red and orange tones indicate areas with high temperatures.

The data demonstrate that the temperature increase is especially pronounced in the Aran zone, the Absheron Peninsula, as well as in the southern region (Lankaran Plain). The increase is relatively weak in mountainous and foothill areas, while spatial differences between the two analysed years are also evident in these regions. Specifically, the minimum annual average temperature for 2020 is -10.2°C, then for 2024, this indicator has already increased to -8.8°C. The maximum annual average temperature also changed significantly during this period. While this indicator was 7.75 degrees in 2020, it increased to 8.13 degrees by 2024. Such temperature differences may be associated with reduced soil moisture availability and increased evaporative demand. Such climatic differences may affect the suitability of typical biotopes for hydrophytic and hygrophytic plants, including representatives of the Cyperaceae family.

The maps presented in Fig. 6 comparatively illustrate precipitation amounts for the years 2020 and 2024. As indicated by the legend, slight changes can be observed between the two analysed years. The observed differences in precipitation between the analysed years may have implications for moisture-dependent habitats, particularly in the Central Aran, Nakhchivan AR, Absheron and Karabakh plains. The highest precipitation levels are observed in the Guba-Khachmaz, Shaki-Zagatala and Lankaran zones, while precipitation differences between the analysed years vary spatially across the country. Such spatial and interannual differences in precipitation may influence moisture availability in wetland and meadow habitats, particularly in areas that are already characterised by relatively low precipitation.

## Discussion

The Republic of Azerbaijan has a very rich flora. There are more than 4,500 species of higher plants here. The flora of Azerbaijan is much richer in terms of the number of species than the flora of other republics of the South Caucasus. Azerbaijan is home to approximately 66% percent of all plant species found across the entire Caucasus (Society (2026), Mammadov 2007).

A conspectus of the family has been compiled, based on a comparative analysis of the Azerbaijani flora, based on classical and modern sources. It is known that classical literature, such as "Flora of Azerbaijan" (Flora of Azerbaijan 1952) and "Flora of the Caucasus" (Grossheim 1940), mentions 115 species of the Cyperaceae family, represented by 20 genera, for Azerbaijan. However, new research has shown that there is significant variation within the family at the genus and species level, and even within taxa that are recogniszed as subspecies.

There are a large number of studies worldwide predicting the significant impacts of anthropogenic factors and climate change on ecosystems and species (Warren et al. 2009). Such studies identify climate change as the primary driver of positive, and more often negative, effects on species and ecosystems. These worldwide studies have been conducted largely independently of each other and provide projections across a wide range of future climate scenarios. This makes it difficult to compare results and obtain a clear and generaliszed picture of how the impacts are occurring due to the increase in global average temperature. The generaliszed picture is important to us for two reasons: in the first direction, research addresses climate change as a global phenomenon; in the second direction, the conducted research allows us to evaluate policy recommendations for the level of temperature increase recommended by the EU (European Union) as a "safe" limit of 2°C.

The high sensitivity of Cyperaceae species to hydrological changes observed in the present study is consistent with findings reported in other regions. Recent studies indicate that species with restricted niches, such as many aquatic Cyperaceae, are highly susceptible to extreme climate variations, which can intensify processes, such as eutrophication and the loss of essential habitats. Furthermore, Cyperaceae species are major or even dominant components of wetland habitats, and the decline of sedge species within these habitats serves as a useful indicator of potential habitat damage. The climatic differences observed between the analysed years in Azerbaijan provide a relevant context for understanding the ecological sensitivity of Cyperaceae to temperature and precipitation conditions. Given the strong dependence of many Cyperaceae species on hydrological conditions, variations in moisture availability may influence their distribution and habitat suitability (Sousa et al. 2025).

The analyses indicate that the observed climatic differences between the analysed years may influence the environmental conditions of plant communities formed by representatives of the Cyperaceae family. The spatial distribution of suitable humid habitats for these species is particularly associated with the Lankaran natural region, river valleys along the Kura-Araz depression, and foothill humid meadows. In the Aran and Absheron regions, where relatively dry conditions prevail, the occurrence of these species may be more dependent on local moisture availability and irrigated habitats. Under relatively dry conditions, Caricetum–Scirpusetum and Eleocharisetum communities may become more vulnerable to changes in moisture availability, potentially favouring drought-tolerant plant communities dominated by representatives of Poaceae Barnhart. and Asteraceae Martinov.

Studies conducted in degraded wetland ecosystems have demonstrated that Cyperaceae coverage decreases significantly with intensifying degradation, while drought-tolerant graminoid taxa increase correspondingly (Sousa et al. 2025). This pattern is relevant to the present study because variations in temperature and moisture availability may influence the distribution and habitat suitability of Cyperaceae communities. The occurrence of suitable humid habitats in areas such as the Lankaran lowland and Kura-Araz river valleys highlights the importance of local moisture conditions for maintaining hygrophytic plant communities.

The research results indicate that plant communities formed by representatives of the Cyperaceae Juss. family are closely associated with hydrological conditions. Variations in temperature and precipitation during the analysed period may influence the environmental conditions and habitat suitability of these communities. The importance of maintaining wetlands and humid biotopes under changing environmental conditions has also been highlighted in broader assessments (FAO 2020, UNEP 2019). The integration of GIS and remote sensing technologies enables the spatial assessment of vegetation distribution in relation to relief and climatic parameters. This approach is consistent with international frameworks for climate-based monitoring (IPPC 2021).

The GIS-based approach employed in the present study enables the spatially explicit assessment of relationships between climatic parameters and vegetation distribution at the national scale, providing a methodological framework that can be replicated and extended in future monitoring programmes. The integration of WorldClim climate data with field-based floristic inventories provides a useful basis for identifying areas where Cyperaceae habitats may be particularly influenced by climatic and environmental conditions. As highlighted by Parra (Parra 2022), the application of remote sensing and GIS technologies in environmental monitoring offers reliable and reproducible tools for assessing vegetation responses to environmental and climatic conditions across diverse landscape types.

The use of WorldClim and OpenTopography data supports the spatial assessment of climatic conditions and altitudinal variation, which is relevant for understanding the distribution of Cyperaceae habitats and biodiversity patterns in the South Caucasus.

## Conclusion

The present study provides the first comprehensive GIS-based ecogeographical assessment of the Cyperaceae family across Azerbaijan, integrating floristic inventory data with remote sensing and climate analysis. A total of 115 taxa belonging to 14 genera were identified, with *Carex*, *Cyperus* and *Schoenoplectus* representing the most species-rich genera. The spatial distribution of these taxa is strongly structured by landscape and hydrological factors, with the highest species concentrations recorded in the Kura-Araz plain, Lankaran lowland and foothill meadows.

Ecological analysis revealed that the family is dominated by mesophytes (58%), followed by hygromesophytes (21%) and hygrophytes (16%), confirming the strong affinity of Cyperaceae representatives for humid and wetland habitats. The predominance of heliophytes (82%) further reflects the open, moisture-rich environments that characterise the primary distribution zones of the family in Azerbaijan. These ecological groupings collectively demonstrate that Cyperaceae species function as reliable phytoindicators of hydrological balance and soil moisture stability.

GIS-based analysis of WorldClim climate data for 2020–2024 revealed spatial and interannual differences in temperature and precipitation across Azerbaijan. Minimum temperatures increased from −10.2°C to −8.8°C, while maximum temperatures increased from 7.75°C to 8.13°C between the analysed years. Differences in precipitation were also observed, particularly in the Aran zone, Absheron and Nakhichevan regions. These climatic variations may influence moisture availability and habitat suitability for wetland and meadow ecosystems supporting Cyperaceae taxa.

The integration of landscape-soil analysis with climatic and spatial data indicates that the distribution of suitable humid habitats for Cyperaceae species is closely associated with local moisture conditions, particularly in the Lankaran natural region and Kura-Araz river valleys. The observed climatic and environmental differences during the 2020–2024 study period may influence the habitat suitability of Caricetum–Scirpusetum and Eleocharisetum plant communities and their relationships with surrounding vegetation. These findings highlight the importance of continued GIS-based monitoring and further long-term observations to better understand changes in Cyperaceae habitats and their relationship with climatic and environmental conditions.
